# Comprehensive geriatric assessment in primary care: a systematic review

**DOI:** 10.1007/s40520-019-01183-w

**Published:** 2019-04-09

**Authors:** James W. Garrard, Natalie J. Cox, Richard M. Dodds, Helen C. Roberts, Avan A. Sayer

**Affiliations:** 1 Radcliffe Department of Medicine, University of Oxford, Oxford, OX1 2JD UK; 2 Academic Geriatric Medicine, Faculty of Medicine, University of Southampton, Southampton, UK; 3 NIHR Southampton Biomedical Research Centre, University Hospital Southampton NHS Foundation Trust and University of Southampton, Southampton, UK; 4 University Hospital Southampton NHS Foundation Trust, Southampton, UK; 5 AGE Research Group, Institute of Neuroscience, Newcastle University, Newcastle upon Tyne, UK; 6 NIHR Newcastle Biomedical Research Centre, Newcastle upon Tyne Hospitals NHS Foundation Trust and Newcastle University, Newcastle upon Tyne, UK; 7 National Institute for Health Research Collaboration for Leadership in Applied Health Research and Care (NIHR CLAHRC) Wessex, University of Southampton, Southampton, UK

**Keywords:** Comprehensive geriatric assessment, Older people, Primary care

## Abstract

**Background:**

Comprehensive geriatric assessment (CGA) involves the multidimensional assessment and management of an older person. It is well described in hospital and home-based settings. A novel approach could be to perform CGA within primary healthcare, the initial community located healthcare setting for patients, improving accessibility to a co-located multidisciplinary team.

**Aim:**

To appraise the evidence on CGA implemented within the primary care practice.

**Methods:**

The review followed PRISMA recommendations. Eligible studies reported CGA on persons aged ≥ 65 in a primary care practice. Studies focusing on a single condition were excluded. Searches were run in five databases; reference lists and publications were screened. Two researchers independently screened for eligibility and assessed study quality. All study outcomes were reviewed.

**Results:**

The authors screened 9003 titles, 145 abstracts and 97 full texts. Four studies were included. Limited study bias was observed. Studies were heterogeneous in design and reported outcomes. CGAs were led by a geriatrician (*n* = 3) or nurse practitioner (*n* = 1), with varied length and extent of follow-up (12–48 months). Post-intervention hospital admission rates showed mixed results, with improved adherence to medication modifications. No improvement in survival or functional outcomes was observed. Interventions were widely accepted and potentially cost-effective.

**Discussion:**

The four studies demonstrated that CGA was acceptable and provided variable outcome benefit. Further research is needed to identify the most effective strategy for implementing CGA in primary care. Particular questions include identification of patients suitable for CGA within primary care CGA, a consensus list of outcome measures, and the role of different healthcare professionals in delivering CGA.

**Electronic supplementary material:**

The online version of this article (10.1007/s40520-019-01183-w) contains supplementary material, which is available to authorized users.

## Introduction

Comprehensive geriatric assessment (CGA) is a multidimensional, multidisciplinary diagnostic and therapeutic process to determine the medical, psychological and functional capabilities of an older person and develop a coordinated and integrated plan for treatment and follow-up [1]. It has been studied intensively and a number of systematic reviews and meta-analyses have shown benefit across healthcare settings [2, 3], including home-based CGA for older people with multimorbidity which has demonstrated reduced hospital admissions and improved mortality rates [4]. It is estimated that a third of the European population will be over 65 by the year 2060 [5] and that worldwide, the number of people aged 80 and above will treble in this time [6]. The effective, holistic management of older people living with multimorbidity and frailty will, therefore, become increasingly necessary [7].

Tools to identify patients at risk of frailty using scoring methods are increasingly used internationally [8, 9] and have recently been integrated into the work of primary care practitioners (PCPs) in the United Kingdom (UK) [10]. The identification of patients with multimorbidity, frailty and complex care needs raises questions about how appropriate provision of assessment and management strategies for this group can be best delivered. Primary healthcare is the community-located healthcare which is the usual first point of contact for patients with healthcare services. In the UK, this typically involves a consultation with a primary care practitioner in a practice (also known as a surgery, clinic or community health centre) but in an appointment that is usually too time limited to undertake CGA. Established avenues for onward referral include community-based services that perform in-home CGA assessment such as community-based geriatric services [11], or secondary care services with review by a geriatrician in an outpatient clinic. There currently appears no established method of assessing these patients within the primary care practice itself. This may be a more appropriate and cost-effective approach for patients who would struggle to attend secondary care but do not require resource-intensive home assessment. Indeed, how best to deliver CGA to older people with multimorbidity in a range of settings was one of the top ten research priorities identified recently by UK priority setting organisation [12]. We, therefore, conducted a systematic review of studies that implemented a CGA in the primary care practice itself. The main aims were to describe the models of CGA implemented, reported outcomes, and acceptability of the intervention compared to existing care.

## Methods

This systematic review was carried out using the methods recommended by the Preferred Reporting Items for Systematic reviews and Meta-Analyses (PRISMA) statement [13]. The study was registered on the International Prospective Register for Systematic Reviews (PROSPERO) Identification number: CRD42016035592.

### Literature search and eligibility criteria

The criteria for study inclusion are presented in Table 1. Articles written in the English language with any design were included if they described a holistic multidimensional assessment (CGA) on persons aged 65 years and over located within the primary care practice. Assessments needed to include direct input by a professional with the generalist skill set required to manage multimorbidity, e.g. PCP, geriatrician or nurse practitioner for older people. The CGA had to be integrated into the primary care practice, namely with the PCP involved in selecting patients likely to benefit and the member of staff performing the intervention having a tangible link to the practice, either an employee or external staff with direct liaison with the PCP. In the case of PCPs undertaking the intervention, it needed to be delivered in a way separate to their usual practice.

**Table 1:** PICO for study inclusion

Population	People aged 65 years and over, not defined by a specific health condition
Intervention	Comprehensive geriatric assessment integrated into the primary care practice
Comparator	Any, or no, comparator used
Outcomes	Primary: method of implementation Secondary: acceptability of the intervention and cost effectiveness. Clinical outcomes of acute care admission, mortality and medicines management

The primary outcome of interest reports on the practical implementation of CGA. Qualitative and quantitative measures on the acceptability of the intervention, and cost effectiveness as well as clinical outcomes including hospital admissions, medication changes and mortality were also of interest.

Studies focused exclusively on a single condition (e.g. diabetes, depression or cancer) were excluded, as the implementation of a model of care focused to one disease is not applicable to management of multimorbidity and the concept of CGA. Studies prior to 2000 were excluded owing to changes in the population structure and healthcare systems, thought not to be applicable to current systems.

The search was run in MEDLINE, EMBASE, Cochrane Library, PsychINFO and CINAHL online databases, with the final search on 1 February 2019. Terms searched were those related to CGA and a primary care setting (an example search strategy is included in ESM Appendix). The reference lists of included publications and citations (identified using MEDLINE) of included studies were screened for relevant articles.

### Data analysis and assessment of risk of bias

Working independently two reviewers (JG, NC) extracted relevant data from included studies. Information extracted included study setting, design and population, patient selection and baseline characteristics, the major processes involved in implementation of CGA and reported outcomes.

The risk of bias of each study was assessed using a set of quantitative criteria outlined by Downs and Black [14] by two reviewers (JG, RD). These criteria provide a quantitative assessment (scored out of 31) of study quality, external validity, internal validity of bias and confounding factors, and power.

This review aimed to highlight key concepts regarding the different methods of implementation of CGA in primary care. Data on patient outcomes and acceptability of the intervention are described and compared in a narrative synthesis as study heterogeneity meant pooling of data for statistical analysis into a meta-analysis was unachievable.

## Results

### Literature search

**Fig. 1:**
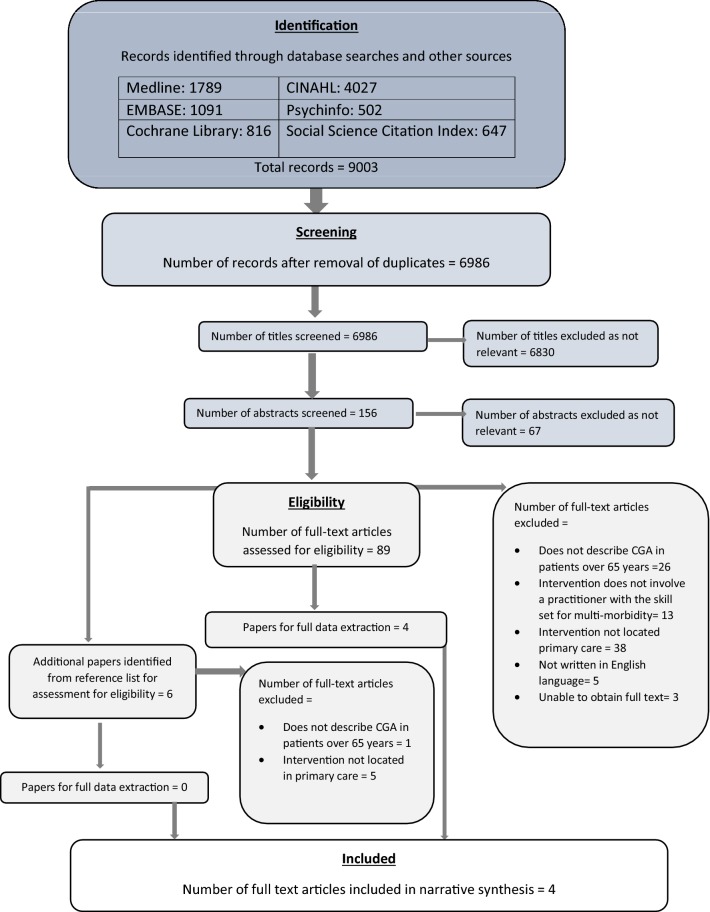
PRISMA flow diagram showing the selection of articles for inclusion and exclusion

Two authors independently screened 9003 titles for relevance to identify 156 abstracts to review (JG, RD). Two authors (JG, NC) reviewed abstracts identifying 95 articles that were assessed for eligibility (including articles identified from reference lists). Attempts were made to obtain more information on the three unavailable full-text abstracts from authors and assess the potential relevance of five articles not available in English. Figure 1 demonstrates the flow diagram of screening articles for eligibility.

Four studies were eligible for inclusion and taken forward to data extraction (JG,NC), totalling 2140 participants (range 186–874 per study) [15–18]. The characteristics of included studies from the United States, Israel and United Kingdom are shown in Table 2. The studies scored between 19 (Hermush [15], Lea [18]) and 24 (Phelan [16]) out of 31 indicative of no major methodological bias (Table 3).

**Table 2:** Details of included studies

Author, year. Country, reference	Subgroup	Study design	Number of participants	Number of practices	Participant mean age (SD) (% female)	CGA intervention				
						Disciplines involved	Initial assessment	Second assessment	PCP involvement	Patient follow-up
Hermush, 2009 Israel [15]	Intervention	Retrospective cohort study	542	5	78 (3) (66%)	Geriatrician, PCP	Geriatrician lead CGA	N/A	Geriatrician proposal (unspecified method)	PCP follow-up with advice as required from geriatrician
Phelan, 2007 United States [16]	Intervention Control	Randomised control trial	433 441	31	81 (5) [65%]	Geriatrician, gerontology ANP, gerontology pharmacist, PCP	ANP lead CGA Pharmacist medication review	ANP and geriatrician clinical review and care plan development with patient	Geriatrician and PCP face-to-face meeting	One follow-up appointment with ANP Ongoing as required telephone follow-up by ANP for ≤ 48 months
Fenton, 2006 United States [17]	Intervention Control	Retrospective matched cohort study	146 437	4	78 (8) [66%]	Geriatrician, PCP	Geriatrician lead CGA	Geriatrician and patient collaborative problem solving	Geriatrician and PCP weekly face-to face meeting and consultation letter	PCP follow-up with advice as required from geriatrician
Lea, 2017 United Kingdom [18]	Intervention	Feasibility study	186	17	Median age 81 (range 65–99) [59%]	Geriatrician, PCP	Geriatrician lead CGA	N/A	Geriatrician and PCP face-to-face meeting at end of session	PCP follow-up Phone advice as required from geriatrician for ≤ 1 year

*CGA* comprehensive geriatric assessment, *ANP* advanced nurse practitioner, *PCP* primary care physician

**Table 3:** Secondary outcome data from included studies

Author of paper	Quality assessment	Comparator to CGA Intervention	Secondary outcomes and results
Hermush [15]	19	Participant baseline (6 months pre-intervention)	Medication management: 68.5% of recommendations implemented (*p*≤0.01)
Phelan [16]	24	Control group receiving usual care	Acceptability of intervention: PCP satisfaction responses − 79% felt improved own management; 80% felt should be present in every clinic Acute care admission: intervention = 18.2% vs. control = 16.4% (*p* = 0.46) Mortality: Intervention (I) 11.4% vs. control (C) 7.1% (*p* = 0.03)
Fenton [17]	20	Control group receiving usual care	Cost–benefit: intervention vs. control −26% lower (*p* = 0.04) Acute care admission: intervention vs. control—conditional IRR 0.57 (*p* = 0.01) Mortality: rate ratio = 1.12 (CI 0.52–2.40)
Lea [18]	19	Participant baseline (6 months pre-intervention)	Acceptability of intervention: PCP and patient satisfaction—positive qualitative feedback Medication management: 6 months 72% of recommendations implemented; by 12 months 65% Acute care admission: IRR 1.83 (CI 1.43–2.34) Mortality: 14.5% at 12 months

Quality of papers as assessed using Downs and Black assessment tool

*CGA* comprehensive geriatric assessment, *PCP* primary care physician

### Study design

There was heterogeneity in study design and participant recruitment across studies. Study designs included one randomised controlled trial [16], one feasibility study [18], one retrospective cohort study [15], and one intervention with retrospectively matched control [17]. The length of follow-up varied from 12 to 48 months [15–18]. Recruitment methods included random selection from the participating PCP’s pool of patients [16], identification of patients with a high level of healthcare utilisation over the preceding 2 years [17], or PCP referral [15–17]. Control populations, when present, received ‘usual clinical care’ [16, 17].

### CGA implementation

All studies described a CGA intervention performed in the primary care clinic or practice and a summary of the models of CGA used is included in Table 2.

The CGA was led in three studies by a geriatrician [15, 17, 18]. Phelan et al. differed with an initial CGA by a gerontology advanced nurse practitioner (ANP), subsequent review of medications by a gerontology pharmacist, and then a second assessment by the ANP with a geriatrician [16]. In all studies, the geriatrician discussed the CGA and management recommendations with the PCP.

The frequency of patient contacts during the CGA intervention varied between studies. Two studies had a scheduled second meeting with a member of the team (ANP or geriatrician) to discuss progress [16, 17]. Ongoing follow-up was then on an ‘as required basis’ for all studies except Lea et al., which did not include patient follow-up as part of the intervention but provided telephone support to PCPs when needed [18].

### Acceptability of interventions

A summary of the secondary outcomes is included in Table 3. The overall satisfaction with the structure of the care model was stated as 'very good' in Phelan et al. [16]. Seventy-one percent of PCPs reported a clear understanding of the intervention, with 79% perceiving improvement in their management of older patients and 80% stating such intervention should be implemented on a larger scale. The authors also reported short-term improvements in geriatric syndrome diagnosis by PCPs at 12 months but this did not persist at 48 months [16].

Qualitative feedback on the acceptability of intervention was positive in Lea et al. [18]. There was perceived clinical benefit by PCPs and patients felt pleased and reassured, with no unfavourable comments [18]. Fenton et al. reported their intervention to be cost effective with a 26% reduction in healthcare costs (*p* = 0.04) [17].

### Clinical outcomes

Three studies reported on hospital admission. Fenton et al. demonstrated a reduction in hospital admission (intervention 20.3/100 person-years vs. 35/100; conditional incidence rate ratio (IRR) 0.57 (*p* = 0.01)) at 48 months compared to control [17]. Phelan et al. demonstrated a non-significant increase in hospital admission at 12 months (intervention 19.4% vs. control 16.2% (*p* = 0.10) and 24 months 18.2% vs. 16.4% (*p* = 0.46) [16]. Lea et al. showed increased rates of admissions 6 months post-intervention compared to pre-intervention, IRR = 1.83 (CI 1.43–2.34) and 1.23 (CI 1.07–1.41), respectively. The following 6 months of follow-up observed stable numbers of unplanned healthcare interactions, reported as a time lag in developing the infrastructure for the CGA process [18]. The lack of a control arm meant the authors were unable to assess if the increase in admissions was related to the CGA intervention [18].

There was no survival improvement in the three studies reporting mortality outcomes [16, 17, 19]; Phelan et al. had significantly higher mortality in the intervention arm at 48 months, 11.4% mortality vs. control: 7.1% (*p* = 0.03) following adjustment for baseline differences.

Drug modification recommendations and ongoing PCP adherence were reported in two studies [15, 18]. Both reported favourable outcomes with adherence to recommendations of 65% at 12 months [18] and 68.5% at 36 months [15], with 61% of recommendations to stop or reduce the dose of medications [15], although there was no control for comparison.

Two studies reported individually on further outcome measures. There was no significant difference in functional ability of participants versus controls at the end of 48 months follow-up observed by Phelan et al. [16]. Hermush et al. reported on the reasons for PCP referral for CGA, with the commonest causes being affective problems (39.7%) and cognitive decline (30.4%). The authors also stated that the mean number of PCP visits pre- to post-intervention dropped from 10.9 to 10.2 (*p* = < 0.01) [15].

## Discussion

This systematic review identified four studies that evaluated a method of implementation of CGA in the primary care practice. All the studies were considered to be low risk of bias. The studies were heterogeneous in their methodology, patient identification and primary outcome data.

A central theme among the included studies was the demonstration of a working relationship between the PCP and geriatrician [15–18] to discuss assessments and onward management for each patient. One review in Australia found that close communication between primary and secondary care providers improved health outcomes and patient satisfaction [20]. The relevant primary care board recommended stronger relationships between service providers with systems to support this, suggesting shared assessments and care plans. The NHS has echoed this by launching plans and legislation in 2015 to improve collaboration and integration of care between NHS services [20–22], and the European Social Protection committee reported on the need to improve the provisions of long-term care throughout Europe [23]. Several such schemes in varying guises have been developed with some suggestion of a reduction in emergency admission rates, although such schemes are often subject to changes in the political landscape [24].

This review has highlighted a focus on the established role of a geriatrician leading to the CGA. Only one study involved CGA led by another healthcare professional [16]. There is recognition that CGA within the community needs development of novel methods alongside research into their efficacy, to address the complex care needs of older people living with multimorbidity or frailty [25]. There is growing interest in the role of other clinicians with specialist expertise such as General Practitioner Extensivists, who use longer patient appointments to undertake holistic clinical assessments [26]. Alternatively, the role of nurse practitioner to perform the CGA, as demonstrated by Phelan [16], is in line with the expanding role of nurses throughout primary care worldwide [27], including consultant practitioners focused on frailty and older persons medicine [16, 28, 29]. Beyond the scope of this review, home-based CGA, often nurse-led [30, 31], provides alternative approaches to primary care-based CGA. Close working relationships between nurses and PCPs enable the development of personalised care plans for frail older individuals and have demonstrated potential improvement in quality of life metrics.

The impact of CGA in primary care on clinical outcomes in these four studies was mixed. Three studies reported variable results for hospital admission rates and no demonstrable improvements in mortality following CGA were seen in three studies [16–18]. One study observed increased mortality; the authors discussed possible causes including that the intervention group may have had a greater severity of illness, or that patients were confused as to who was making decisions on their care and, therefore, less compliant with management [16]. This identifies the importance of clear communication between healthcare professionals and patients and patients when multiple people are involved, particularly when the structure of care is changed. One way to overcome this is to fully support patients to become actively involved in their care, with potential benefits to patients and healthcare providers, including better communication, highlighted in a recent European Commission report [32].

Adherence to prescription modifications, often dosage reduction or cessation of the drug, was the primary outcome in two studies. This remained high until 36 months from initial assessment, suggesting that CGA in primary care may positively contribute to reducing the polypharmacy burden in older people [15, 18], which forms part of the National Institute for Clinical Excellence (NICE) guidance [33]. Importantly, when included in the analysis, CGA interventions were found to be acceptable for PCPs and there was evidence of potential cost effectiveness [16, 18]. This is in keeping with a UK study which focused on implementation of a multi-domain assessment tool in primary care, suggesting that primary care-based assessments may have financial and practical viability [34].

### Strengths and limitations of the review

We conducted a rigorous systematic review following the PRISMA guidelines, including the use of two independent reviewers at each stage of the process. At each stage, if any papers created disagreement, the reviewers met to review the paper and reach consensus. The four eligible papers were also judged to have low risk of bias.

The lack of eligible studies is a major limitation and makes it difficult to draw conclusions around efficacy for methods of CGA implemented in a primary care practice. We did not review the grey literature and, therefore, there may be service development or quality improvement initiatives that could provide further insight. Five papers not written in the English language were also excluded; these may have provided further insights into model of CGA in the primary care setting in other countries.

Given that a model of CGA in primary care requires complex interventions in health and social care delivery, we could also have performed this review as a ‘realist review’. This may have provided greater understanding of the theoretical frameworks behind the interventions, to give a greater understanding of the processes required to implement them [35].

The applicability of evidence found in this review may be difficult to interpret on a wider scale. The structure of the healthcare systems varies greatly across the three countries included. Differing processes, such as choice of primary care clinics, PCPs and other structures available in the community, as well as the role of private healthcare infrastructure, may limit reproduction of implementation strategies in other countries. Study designs varied greatly, limiting the comparability of the observed results between studies.

### Recommendations for future research

Further research is needed to identify the most effective strategy for implementing CGA in primary care. Particular questions of interest include identification of patients most suitable for a CGA within the primary care setting, a consensus list of outcome measures, and the role of different healthcare professionals in delivering CGA. These areas would also benefit from robust health economic evaluation.

## Conclusion

This systematic review identified only four studies that described the implementation of CGA in a primary care practice, as opposed to hospital or home setting. The evidence in these heterogeneous studies indicated that CGA based on the primary care practice was acceptable to those involved, but with variable impact on the outcomes measured. In a small sample, potential benefits include cost effectiveness, improved medication adherence and reduced hospital admission rates. This may reflect methodological differences in the studies and variations in the health systems of the three countries where they were conducted. Mortality outcomes were inconsistent. The main potential negative effect of practice-based CGA may be in creating confusion as to the ‘ownership’ of a patient’s management and highlights the need for communication with patients, to improve compliance and prevent the risk of harm. Primary care would be a natural setting for CGA to identify and support the majority of people with multimorbidity and frailty and further research is warranted.

## Electronic supplementary material

Below is the link to the electronic supplementary material.

Supplementary material 1 (DOCX 16 kb)
