# Supplementary material for: Comprehensive geriatric assessment in primary care: a systematic review
Source: Aging Clin Exp Res. 2019 Apr 9;32(2):197–205. doi: 10.1007/s40520-019-01183-w (PMC7033083; doi:10.1007/s40520-019-01183-w)
Supplement: Supplementary file 1 — Supplementary material 1 (DOCX 16 kb) [file 40520_2019_1183_MOESM1_ESM.docx]

**Appendix 1: search terms for databases:**

## Search terms:

Medline Search Strategy Via OVID SP

1. exp Aged/

2. elder*.tw.

3. aged.tw.

4. old*.tw.

5. geriatric*.tw.

6. 1 or 2 or 3 or 4 or 5

7. Primary Health Care/

8. exp General Practice/

9. (primary adj3 care).tw.

10. family practi*.tw.

11. general practi*.tw.

12. 7 or 8 or 9 or 10 or 11

13. 6 and 12

14. (comprehensive adj3 assessment).tw.

15. Geriatric Assessment/

16. Needs Assessment/

17. 14 or 15 or 16

18. 13 and 17

19. limit 18 to (english language and yr="2000 -Current")

EMBASE Search Strategy via OVIDSP

1. exp aged/

2. elder*.tw.

3. aged.tw.

4. old*.tw.

5. geriatric*.tw.

6. 1 or 2 or 3 or 4 or 5

7. exp primary health care/

8. general practice/

9. (primary adj3 care).tw.

10. family practi*.tw.

11. general practi*.tw.

12. 7 or 8 or 9 or 10 or 11

13. 6 and 12

14. geriatric assessment/

15. needs assessment/

16. (comprehensive adj3 assessment).tw.

17. 14 or 15 or 16

18. 13 and 17

19. limit 18 to (english language and yr="2000 -Current")

Cochrane search term

1. exp aged/

2. elder*.tw.

3. aged.tw.

4. old*.tw.

5. geriatric*.tw.

6. 1 or 2 or 3 or 4 or 5

7. exp primary health care/

8. general practice/

9. (primary adj3 care).tw.

10. family practi*.tw.

11. general practi*.tw.

12. 7 or 8 or 9 or 10 or 11

13. 6 and 12

14. geriatric assessment/

15. needs assessment/

16. (comprehensive adj3 assessment).tw.

17. 14 or 15 or 16

18. 13 and 17

19. limit 18 to (english language and yr="2000 -Current")

Psychinfo search terms

- S22 S14 AND S21
- S21 S15 OR S16 OR S17 ORS18ORS19OR
- S20
- S20 (MH "Community Health Services")
- S19 (MH "Health Services for the Aged")
- S18 multidisciplinary assessment
- S17 (MH "Multidisciplinary Care Team")
- S16 (MH "Primary Health Care") OR (MH "Physicians, Family") OR "primary care"
- S15 (MH "Family Practice") OR "general practice"
- S14 S6 AND S13
- S13 S7ORS8ORS9OR S10 OR S11 OR S12
- S12 (MH "Patient Assessment")
- S11 (MH "Needs Assessment")
- S10 needs assessment
- S9 "physical health assessment"
- S8 comprehensive geriatric assessment
- S7 (MH "Geriatric Functional Assessment") OR (MH "Geriatric Assessment") OR (MH "Clinical Assessment Tools")
- S6 S1ORS2ORS3OR S4ORS5
- S5 geriatric*
- S4 old*
- S3 elder*
- S2 (MH "Aged")
- S1 aged

CINAHL search terms

- S22 S14 AND S21
- S21 S15 OR S16 OR S17 ORS18ORS19OR
- S20
- S20 (MH "Community Health Services")
- S19 (MH "Health Services for the Aged")
- S18 multidisciplinary assessment
- S17 (MH "Multidisciplinary Care Team")
- S16 (MH "Primary Health Care") OR (MH "Physicians, Family") OR "primary care"
- S15 (MH "Family Practice") OR "general practice"
- S14 S6 AND S13
- S13 S7ORS8ORS9OR S10 OR S11 OR S12
- S12 (MH "Patient Assessment")
- S11 (MH "Needs Assessment")
- S10 needs assessment
- S9 "physical health assessment"
- S8 comprehensive geriatric assessment
- S7 (MH "Geriatric Functional Assessment") OR (MH "Geriatric Assessment") OR (MH "Clinical Assessment Tools")
- S6 S1ORS2ORS3OR S4ORS5
- S5 geriatric*
- S4 old*
- S3 elder*
- S2 (MH "Aged")
- S1 aged

Web of science search terms:

- #25 AND #20 Refined by: LANGUAGES: ( ENGLISH ) Timespan=All years Search language=Auto
- #25 AND #20
- #24 OR #23 OR #22 OR #21
- TOPIC: (community health) Timespan=All years Search language=Auto
- TOPIC: (family practice) Timespan=All years Search language=Auto
- TOPIC: (general practice) Timespan=All years Search language=Auto
- TOPIC: (primary health care) Timespan=All years Search language=Auto
- #19 AND #18
- #17OR#16OR#15OR#14OR#13OR#12OR#11OR#10OR#9OR#8
- #8OR#7OR#6OR#5OR#4OR#3OR#2OR#1
- TOPIC: (needs assessment) Timespan=All years Search language=Auto
- TOPIC: (geriatric management) Timespan=All years Search language=Auto
- TOPIC: (health services for the aged) Timespan=All years Search language=Auto
- TOPIC: (geriatric health services) Timespan=All years Search language=Auto
- TOPIC: (needs assessment) Timespan=All years Search language=Auto
- TOPIC: (multi-domain intervention) Timespan=All years Search language=Auto
- TOPIC: (multidisciplinary assessment) Timespan=All years Search language=Auto
- TOPIC: (multidisciplinary team assessment) Timespan=All years Search language=Auto
- TOPIC: (geriatric assessment) Timespan=All years Search language=Auto
- TOPIC: (elderly care) Timespan=All years Search language=Auto
- TOPIC: (old*.tw) Timespan=All years Search language=Auto
- TOPIC: (elder*.tw) Timespan=All years Search language=Auto
- TOPIC: (geriatric) Timespan=All years Search language=Auto
- TOPIC: (frail adults) Timespan=All years Search language=Auto
- TOPIC: (older adults) Timespan=All years Search language=Auto
- TOPIC: (elderly) Timespan=All years Search language=Auto
- TOPIC: (aged) Timespan=All years Search language=Auto
